# Prevalence of Coronal Pulp Stones and Its Relation with Systemic Disorders in Northern Indian Central Punjabi Population

**DOI:** 10.1155/2014/617590

**Published:** 2014-04-22

**Authors:** Sandeep Kumar Bains, Archana Bhatia, Harkanwal Preet Singh, Swati Swagatika Biswal, Shashi Kanth, Srinivas Nalla

**Affiliations:** ^1^Department of Oral Medicine and Radiology, Dasmesh Institute of Research and Dental Sciences, Faridkot, Punjab, India; ^2^Department of Periodontics, Dasmesh Institute of Research and Dental Sciences, Faridkot, Punjab, India; ^3^Department of Oral Pathology and Microbiology, Dasmesh Institute of Research and Dental Sciences, Faridkot, Punjab, India; ^4^KLES Institute of Dental Sciences, Bangalore, Karnataka, India; ^5^Department of Maxillofacial Surgery, Azamgarh Dental College, Azamgarh, Uttar Pradesh, India; ^6^Department of Orthodontics and Dentofacial Orthopedics, Al Badar Rural Dental College and Hospital, Naganhalli Road, Daryapur, Gulbarga, India

## Abstract

*Aim*. To estimate the prevalence of coronal pulp stones in the molar teeth of dental outpatients of Sunam, Sangrur district, Punjab, India, to report any association between occurrence of pulp stones with age, gender, dental arch, side, and dental status and to find out correlation between pulp stones with dental and systemic diseases. *Materials and Methods*. 500 routine dental outpatients within age group of 18–67 years were involved in the study. Molar bitewing of left and right side of each patient was taken with XCP bitewing instrument and size 2 film. The presence or absence of pulp stones was recorded. Chi-square analysis was used to record the prevalence of pulp stones and to compare it with demographic and systemic factors. *Results*. Overall prevalence of pulp stones was 41.8%. Pulp stones were significantly higher in maxilla (11.59%) than mandible (6.54%), left side than right side, and first molar than other molars. Higher numbers of pulp stones were recorded in patients with cardiovascular disease (38.89%) than with cholelithiasis and renal lithiasis. *Conclusion*. Pulp stones were higher in maxillary arch than mandibular arch and in females than males. Cardiovascular patients had higher number of pulp stones than other groups.

## 1. Introduction

Pulp stones are foci of calcification in the pulp of tooth. Calcification can occur in the dental pulp as discrete calcified stones or as diffuse form that can occur freely in the pulp tissue or is attached to or embedded into dentin [1]. Depending on their microscopic structures, pulp stones have been classified into true or false form. They are not clinically apparent but are common radiographic findings [2].

They have variable radiographic appearance; they may be radiopaque structure within the pulp chamber or in the root. They do not have uniform shape or number. They may be round or oval, and some pulp stones inhabit most of the pulp chamber. Some may be large as 2 or 3 mm in diameter. Only these large calcified concretions are radiographically discernible. Pulp stones occur most commonly in molars, although they occur in all tooth types [2]. Healthy, deceased, and even unerupted teeth can have pulp stones [3]. Half the teeth of young people and in almost all the teeth of people older than fifty years of age have pulp stones which are probably apparent microscopically [2]. Pulp degeneration, inductive interactions between epithelium and pulp tissue, age, circulatory disturbances in the pulp, nanobacteria [4], orthodontic tooth movements, idiopathic factors, genetic predisposition [1], fluoride supplementation [5], and Marfan syndrome [6] are the few factors which are implicated in pulp stones formation. Their formation may be associated with long standing irritants such as caries, deep fillings, and chronic inflammation. Some authors suggest that pulp stones are a feature of an irritated pulp, attempting to repair itself [1].

Pulpal pain is one of the frequent symptoms associated with pulp stones. The pain may vary from mild to severe [4, 7]. They can cause obstruction of the root canals which leads to endodontic failure [8]. Calcific atheromas and the calcification of dental pulp may have a similar pathogenesis so the routine dental radiographs may be useful as a rapid screening method for early identification of potential cardiovascular diseases. So, oral and maxillofacial radiology may be helpful in screening for cardiovascular diseases [9].

The present study aimed at estimating the prevalence of pulp stones by bitewing radiographs. This study also aimed at correlating the prevalence of pulp stones with that of age, gender, dental status, dental diseases, and systemic diseases.

## 2. Materials and Method

This study was conducted in the Department of Oral Medicine and Radiology, Guru Nanak Dev Dental College, Hospital & Research Institute, Sunam, over an 18-month period. 500 routine dental outpatients within age group of 18–67 years were involved in the study. Ethical permission was taken before the commencement of study. Patients with grossly destructed teeth, teeth with metal crowns, and extensive metallic restoration and poor quality radiographs were excluded from study sample.

**Figure 1:**
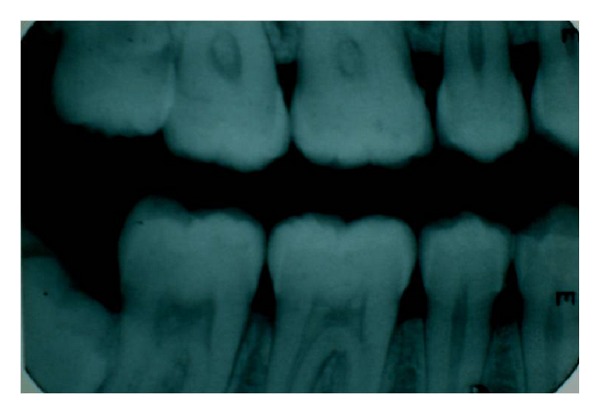
Pulp stones in 16, 17, 46, and 47 teeth.

**Figure 2:**
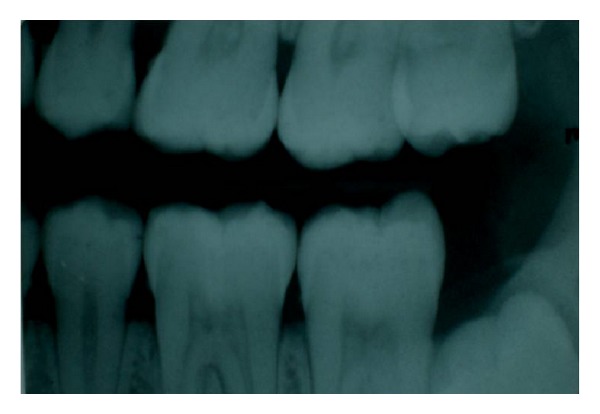
Pulp stones in 26, 27, and 36 teeth.

**Figure 3:**
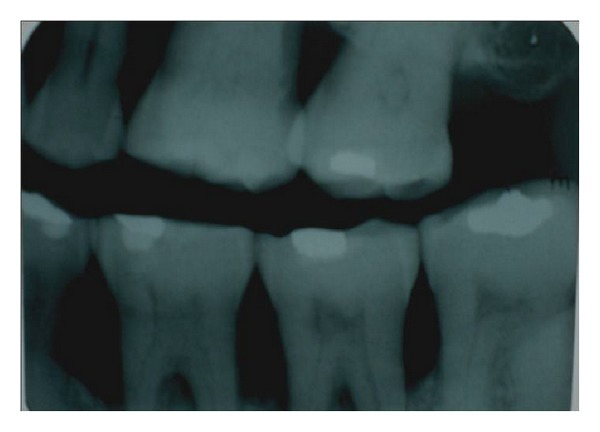
Pulp stones in attrited, restored, and periodontically involved teeth.

**Figure 4:**
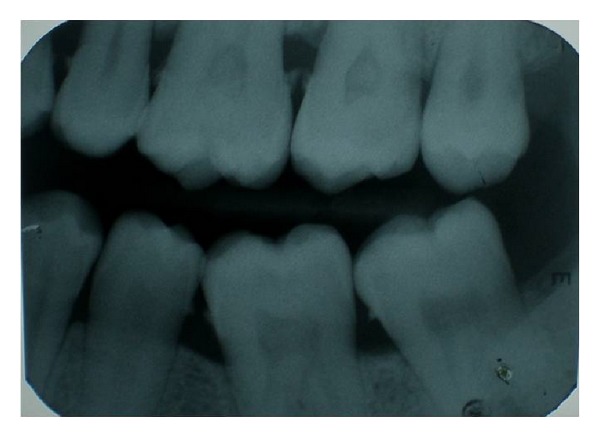
Pulp stones in periodontically involved teeth.

**Figure 5:**
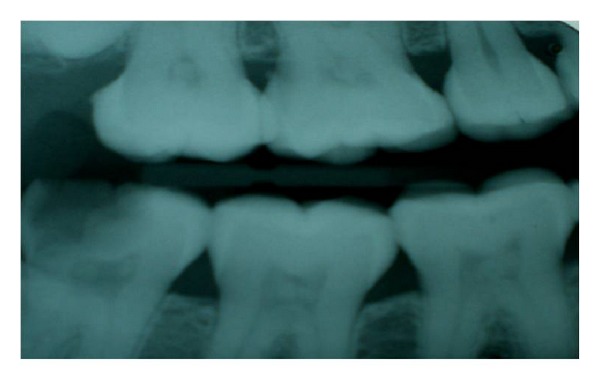
Pulp stones in carious teeth.

The patients were informed regarding the study and an informed written consent was obtained. A case history Performa was designed to obtain patient information regarding age, sex, periodontal status, history of orthodontic treatment, dental status (caries, restoration, attrition), and systemic diseases. Patients were divided into 5 age groups of 100 each, that is, 18–27 years, 28–37 years, 38–47 years, 48–57 years, and 58–67 years. Patients were made to wear lead apron and thyroid collar and sit on the chair. Head rest was adjusted to support and position the patient's head so that the upper arch is parallel to the floor and mid-sagittal plane is perpendicular to the floor. The extension cone paralleling (XCP) bitewing instrument was assembled in patient's mouth with film and the patient was asked to bite on the bite block. Tube head was adjusted +10 degree to the external guide ring to make the beam parallel with the occlusal plane maintaining the 16-inch of focal spot to object distance. Molar bitewing radiographs of right and left side of each patient were taken using intraoral radiographic unit operating at 70 kilovoltage peak and 8 milliamperes by standard exposure parameters. Films were exposed. Exposed films were manually processed under standardized processing conditions in light proof dark room and were dried. Dried films thus obtained were viewed by using X-ray viewer and magnifying glass for the presence or absence of pulp stones (Figures 1, 2, 3, 4, and 5). Data obtained was tabulated and statistically analyzed with application of Statistical Package for the Social Sciences (SPSS) version 5.0 using chi-square test and Fisher's exact test.* P* value less than 0.05 was considered statistically significant.

## 3. Results

Overall prevalence of pulp stones in both the gender was 41.8% (209/500) and in teeth was 9.09%. Out of 257 males, 98 had pulp stones and out of 243 females, 111 had pulp stones. Pulp stones were significantly higher in maxilla than mandible (Max. = 11.59%, Mand. = 6.54%) (Table 1) (Figure 1). Pulp stones were higher in left side than right side and they were significantly higher in 26 (21%). Pulp stones were higher in the first molar than the second and third molar (Table 2). Pulp stones were significantly higher in 17, 26, 37, and 46 of females than males. The age group from 58–67 years showed higher pulp stones as compared to other groups (46.05%); difference was statistically nonsignificant (Table 3). The prevalence of pulp stones in attrited teeth was (male = 12.19%, female = 26.22%) 18.18% (Tables 4 and 5).

The prevalence of pulp stones in periodontal pathology teeth (male = 15.78%, female = 17.20%) was 16.41%. The prevalence of pulp stones in carious teeth (male = 5.22% female = 7.32%) was 6.23%. The prevalence of pulp stones in restored teeth (male = 13.55%, female = 10.96%) was 12.34% (Tables 4 and 5).

The prevalence of pulp stones in orthodontically treated patients (male = 6.25%, female = 7.14%) was 6.66% (Table 6). The prevalence of pulp stones in arteriosclerotic (male = 50%, female = 25%) patients was 38.88%. The prevalence of pulp stones in renal stone (male = 20% and female = 14.2%) patients was 16.66%. The prevalence of pulp stones in cholelithiasis (male = 0% and female = 25%) patients was 10% (Table 7).

## 4. Discussion

The present study comprised of 500 patients, 243 females and 257 males within age group of 18–67 years. Molar bitewing radiographs of right and left side of each patient were taken and evaluated by maxillofacial radiologist for presence of pulp stones.

The prevalence of pulp stone calculated in this study was 41.8% and females exhibited higher pulp stones than male and maxillary teeth had higher pulp stones than mandibular teeth which are in accordance with other studies conducted by Ranjitkar et al. [1], Tamse et al. [10], and Goga et al. [11]. The prevalence of pulp stones in this study was found to be higher in the first molar than in the second molar which is in agreement with other investigators [1, 10, 12]. A plausible explanation is that the early eruption of the first molar will expose them for long period of time, to more degenerative changes, thus confirming that the calcification of the pulp increases with the time [13]. Al-Nazhan and Al-Shamrani [13] concluded that most attributable reason could be that as age advances the structure of the normal pulp varies. This usually leads to a progressive decrease in the number of pulp cells as well as gradual increase in mucopolysaccharides and fibrous elements leading to calcification. In the present study, 58–67 years group showed higher pulp stones which was in harmony with Sayegh and Reed study [14].

Our study showed that 16.41% teeth with pulp stones were associated with periodontal pathology. Reports dealing with the effect of periodontal disease on the pulp tissue showed a close relationship between the presence of pulp calcifications and periodontal disease. Sheykhrezaee et al. concluded that periodontal disease can lead to fibrosis and calcification [15]. Sübay et al. examined sixty teeth with various degrees of periodontal disease and found pulp calcification in 78% of teeth and suggested that periodontal disease interferes with blood supply and nutrition of the pulp causing decrease in cellular elements and increase in calcification [16].

The present study revealed that out of 143 attrited teeth, 26 teeth showed pulp stones which were less than reported by Al-Nazhan and Al-Shamrani [13]. Studies have shown that irritants like attrition and caries can lead to deleterious influence on the pulp [9]. Spouge reported that the physical abrasiveness of the diet and the highly developed muscles of mastication account in part for the high rate of attrition. Irritation in form of attrition causes circulatory disturbances and thrombosis which mineralizes leading to pulp stone formation [13].

Carious lesions stimulate inflammatory changes within pulp leading to secondary dentin formation and increased calcification [5]. The recent literature suggests that pulp stones are a feature of an irritated pulp, an attempt to repair itself [1]. In our investigation we observed that out of 481 carious teeth, 30 (6.2%) teeth showed pulp stones. However, prevalence was low as noticed by Al-Nazhan and Al-Shamrani [13]. It is known that trauma in the form of restorative procedure can cause capillary thrombosis and/or vascular wall damage which on mineralization can lead to formation of pulp stone. In this study, out of 332 restored teeth, 41 (12.3%) teeth had pulp stones. This was in accordance to Al-Nazhan and Al-Shamrani.

Sayegh and Reed [14] concluded that systemic variations such as arteriosclerosis and renal lithiasis can be considered as factors predisposing to pulpal calcification which was later confirmed by Moura and Paiva in his radiographic study. Edds et al. found a significant (75%) relationship between preexisting cardiovascular disease and pulp stones. In our study, out of 18 arteriosclerotic patients, 7 (38.8%) had pulp stones which is less than that reported by Edds et al. [9].

Out of 12 renal stone patients, 2 (16.67%) had pulp stones and out of 10 cholelithiasis patients 1 had pulp stone (10%). Stafne and Szabo [17] suggested that pulp stones are not directly responsible for the production of renal stones and gall stones. However, Çiftçiouglu et al. proposed that nanobacteria may induce pulp calcification and kidney stone and gall stone formation [4].

So, we suggest that patients with pulp stones have high propensity to develop cardiovascular or cholelithiasis. So, such patients should be screened to asses such complications at early stages. Orthodontic force application may produce periodontal inflammatory reaction. Forces can cause odontoblastic layer degeneration due to circulatory disturbances in human pulp tissue causing calcification. The present study showed that two patients (6.66%) had pulp stones out of 30 orthodontically treated patients. Delivanis found 2 patients (4.34%) having pulp calcification out of 46 orthodontic treated patients. Sübay et al. [16] found 17.5% pulp stones in patients undergoing orthodontic treatment. They concluded in their study that extrusive forces applied to teeth do not cause significant pathological changes in human pulp tissue.

## 5. Conclusion

Considering the fact that this is the first study in Punjabi population that may provide a preliminary data regarding the usefulness of bitewing radiography for coronal pulp stone estimation and their implication in endodontic treatment and its relationship with pain, this study may be used as a rapid screening method for early identification of potential cardiovascular diseases. It may serve as an adjunct in forensic odontology. However, large scale longitudinal studies are required to substantiate the findings obtained in this study.

## Figures and Tables

**Table 1:** Prevalence of pulp stones and dental arches.

	Maxilla	Mandible	*P* value
Total teeth	2690	2643	0.001
Pulp stones	312	173	
Percentage (%)	11.59	6.54	

**Table 2:** Prevalence of pulp stones and tooth type.

Tooth type	Total teeth	Pulp stones	Percentage	*P* value
16	468	78	16.7%	0.0001
17	472	53	11.2%	
18	394	7	1.8%	
26	471	99	21%	
27	483	67	13.87%	
28	402	8	2%	
36	440	40	9.09%	
37	452	34	7.5%	
38	424	6	1.4%	
46	440	51	11.6%	
47	459	33	7.2%	
48	428	9	2.1%	
Total	**5333**	**485**	**9.09%**	

**Table 3:** Prevalence of pulp stones in relation to age group of males and females.

Age groups (years)	Total patients = 500				*P* value
	Female		Male		
	Total patients	Patient with pulp stones	Total patients	Patient with pulp stones	
18–27	48	14 (29.2%)	52	25 (48.1%)	0.05
28–37	45	16 (35.6%)	55	26 (47.3%)	
38–47	51	24 (47.1%)	49	19 (38.8%)	
48–57	58	22 (37.9%)	42	18 (42.9%)	
58–67	55	22 (40.0%)	45	23 (51.1%)	
Total	**257**	**98 (38.1%)**	**243**	**111 (45.7%)**	

**Table 4:** Prevalence of pulp stones and dental status in males (max. and mand. arch).

Arch	Dental status									*P* value
	Tooth type	Attrition		Periodontal pathology		Carious		Restored		
		Total teeth	PS	Total teeth	PS	Total teeth	PS	Total teeth	PS	
Max.	1st M	21	3 (14.2)	147	37 (25.2)	37	2 (5.4)	24	6 (25)	0.37
	2nd M	13	1 (7.6)	100	21 (21)	36	3 (8.3)	25	6 (24)	
	3rd M	7	1 (14.2)	24	1 (4.1)	24	1 (4.1)	13	1 (7.76)	
Mand.	1st M	17	3 (17.6)	147	18 (12.2)	58	4 (6.8)	41	4 (9.7)	
	2nd M	15	1 (6.6)	143	15 (10.4)	47	2 (4.2)	50	6 (12)	
	3rd M	9	1 (11.1)	28	1 (3.5)	47	1 (2.1)	24	1 (4.1)	

**Table 5:** Prevalence of pulp stones and dental status in females (max. and mand. arch).

	Dental status									*P* value
	Tooth type	Attrition		Periodontal pathology		Carious		Restored		
		Total teeth	PS	Total teeth	PS	Total teeth	PS	Total teeth	PS	
Max.	1st M	14	4 (26.8)	106	25 (23.6)	34	4 (11.8)	29	4 (13.7)	0.23
	2nd M	10	2 (20)	88	21 (23.8)	34	2 (5.8)	14	2 (14.2)	
	3rd M	7	1 (14.2)	26	2 (7.7)	30	2 (6.6)	7	1 (14.2)	
Man.	1st M	14	5 (35.7)	117	19 (16.2)	64	4 (6)	40	8 (20)	
	2nd M	13	3 (23)	103	12 (11.6)	31	3 (9.6)	43	1 (2)	
	3rd M	5	1 (20)	25	1 (4)	39	2 (5)	22	1 (4.5)	

**Table 6:** Prevalence of pulp stones in orthodontically treated patients.

Orthodontically treated patients			*P* value
	Total	Pulp stones	
Male	16	1 (6.25)	1
Female	14	1 (7.1)	

**Table 7:** Prevalence of pulp stones and systemic diseases.

	Systemic diseases						*P* value
	Atherosclerosis		Renal stones		Cholelithiasis		
	Total	Pulp stones	Total	Pulp stones	Total	Pulp stones	
Male	10	5 (50%)	5	1 (20%)	6	0	0.367
Female	8	2 (25%)	7	(14.2)	4	1 (25%)	
